# Predictive Modeling of Thiol Changes in Raw Ground Pork as Affected by 13 Plant Extracts—Application of Arrhenius, Log-logistic and Artificial Neural Network Models

**DOI:** 10.3390/antiox10060917

**Published:** 2021-06-05

**Authors:** Małgorzata Muzolf-Panek, Anna Kaczmarek

**Affiliations:** Department of Food Quality and Safety Management, Faculty of Food Science and Nutrition, Poznan University of Life Sciences, Wojska Polskiego 31, 60-637 Poznań, Poland; anna.kaczmarek@up.poznan.pl

**Keywords:** thiol content, protein oxidation, raw pork, plant extracts, predictive models, temperature effect, Arrhenius equation, log-logistic model, artificial neural network

## Abstract

In this study, predictive models of protein oxidation, expressed as the content of thiol groups (SH), in raw ground pork were established and their accuracy was compared. The SH changes were monitored during, maximum, 11 days of storage at five temperature levels: 4, 8, 12, 16, and 20 °C. The effect of 13 plant extracts, including spices such as allspice, black seed, cardamom, caraway, cloves, garlic, nutmeg, and onion, and herbs such as basil, bay leaf, oregano, rosemary, and thyme, on protein oxidation in pork was studied. The zero-order function was used to described SH changes with time. The effect of temperature was assessed by using Arrhenius and log–logistic equations. Artificial neural network (ANN) models were also developed. The results obtained showed very good acceptability of the models for the monitoring and prediction of protein oxidation in raw pork samples. High average R^2^ coefficients equal to 0.948, 0.957, and 0.944 were obtained for Arhhenius, log-logistic and ANN models, respectively. Multiple linear regression (MLR) was used to assess the influence of plant extracts on protein oxidation and showed oregano as the most potent antioxidant among the tested ones in raw ground pork.

## 1. Introduction

Meat is a valuable source of protein, containing essential amino acids, heme-iron, B vitamins, and minerals such as zinc and phosphorus [1], and pork meat is one of the most popular meat types worldwide [2]. Although vegetarian or vegan lifestyles have become popular, mostly in high-developed countries, their impact on global changes in consumers’ preferences is still relatively low and the global consumption of meat is maintained at a high level and is predicted to increase for pig meat to 127 Mt over the next 10 years. This increase will be the most noticeable in developing countries (such as Latin America), where the consumption of pork is expected to boost [2].

During shelf life, meat undergoes a number of processes that negatively affect the final quality and safety of the product. Lipid oxidation has been considered for a long time to be the main process leading to the decrease in the sensory and nutritional values of meat [3,4]. Starting two decades ago, increasing attention has been paid to proteins as the target of oxidation [5,6,7]. The oxidation reactions in protein fraction could be initiated indirectly by the primary and secondary products of lipid oxidation [4,5]. It was reported that both lipid and protein oxidation in meat processes are very complex and inextricably related to each other [8,9,10]. Protein oxidation could be also induced directly by the reactions with reactive oxygen species (ROS) or transition metals [5,6]. There are various final products of protein oxidation depending on the target of the process, protein structure, presence of amino acids such as cysteine and methionine, oxidizing system (ROS or other), and some intrinsic (presence of endogenous antioxidants and prooxidants) and extrinsic conditions (meat processing conditions, etc.) [4,7,11]. Generally, oxidative modifications of the proteins include cleavage in the backbone position, leading to protein fragmentation; oxidative modification of sulfur-containing amino acids (cysteine and methionine), resulting in the loss of thiol groups and disulfide bond formation; intra- and inter-molecular cross-linkage; and carbonyl generation [6,7,11]. All these reactions could take place through various pathways that could cause changes in the protein conformation, digestibility, and solubility and loss of essential amino acids, and affect the sensory and nutritional values and the technological properties of the meat [5,12]. In addition, minced meat is particularly susceptible to oxidation through exposure to oxygen during the mincing process itself and through the increased surface contact with oxygen.

Various strategies have been undertaken to counteract quality impairments in meat during storage. Consumers expect innovations in this aspect to lead to the decline of so-called “artificial” food (the term used often to describe food with preservatives). Therefore, the “natural” and “clean label” claims are recently widespread marketing catchwords for food without any additives and/or with naturally occurring functional components such as herbs and spices or extracts [13,14]. Thus, the efforts of the food industry and scientists have been focused on replacing chemical preservatives with plant extracts rich in antioxidants and other bioactive compounds. This approach has been met with consumer acceptance, even though the sensory attributes of such “natural” food have been changed in terms of taste, color, and odor [8]. The active antioxidant compounds found in spices and herbs are phenolic acids such as protocatechuic, ferulic, hydroxybenzoic, caffeic, and rosmarinic acids; flavonoids such as kaempferol, quercetin, catechin, and rutin; vitamins; terpenoids found in plant oils (such as tymochinon, eugenol, and carvacrol); and enzymes or glutathione [8,15,16,17,18,19,20,21]. The broad range of bioactive compounds results in various mechanisms of antioxidant action in food, such as free radical scavenging, scavenging molecular oxygen, chelating transition metal ions, regeneration of other antioxidants, and also inhibition of pro-oxidant enzymes [22].

So far, many studies have shown the positive effect of plant and plant-extract addition to meat on the oxidative status of its protein [23,24,25,26,27,28]. However, a predictive approach to protein oxidation in raw meat is still little described [29]. 

Among various external factors, temperature and time of storage were shown to be crucial to the final quality of meat [30]. Thus, it seems reasonable to use the predictive approach (kinetic modeling) to monitor and manage meat-quality changes during shelf life. This tool is very helpful in ensuring and maintaining meat safety, which is an important issue from the producer’s and consumer’s points of view. Kinetic modeling could also be used as an initial step in food-product development [30].

Previously, mathematical models were developed based on the changes of numerous quality attributes in food [29,31,32,33,34,35,36,37,38,39]. ANN models were also previously built for quality assessment of shrimp, bream fillets, and pork-protein denaturation [35,40,41].

To the authors’ best knowledge, there is neither research on the kinetic approach in terms of protein oxidation in pork nor data available on the kinetic modeling of meat quality after plant-extract addition.

Therefore, the goal of this study is to establish predictive models of protein oxidation (expressed as SH content) of raw ground pork with the addition of various plant extracts during storage at different temperatures. The models were derived based on a zero-order kinetic model combined with Arrhenius and log-logistic equations. Data mining techniques such as ANN were also applied for the SH content prediction at various time/temperature conditions. In order to range the extract studied according to its antioxidant effectiveness in raw meat, MLR models were also calculated. 

## 2. Materials and Methods

### 2.1. Materials

All herbs (basil, bay leaf, oregano, rosemary, and thyme) and spices (allspice, black seed, cardamom, caraway, cloves, garlic, nutmeg, and onion) were bought from a local distributor (Ciecierzyn, Poland). Pork neck was supplied by a local producer (Swarzędz, Poland). The meat was minced (diameter of plate = 5 mm) on the day of transport to the laboratory. The temperature during the transport was held at a level of 4–8 °C. The basic composition of the meat was as follows: moisture—64.7%, protein—19.6%, and fat—13.6%.

### 2.2. Preparation and Characterization of Plant Extract

Extracts of spices and herbs were prepared in 50% aqueous ethanol as previously described [8]. The mass (g) to liquid (ml) ratio of each extract was 1:15 (*m*/*v*). The DPPH^•^ radical scavenging capacity was determined by the method by Sánchez-Moreno et al. [42], modified as described previously [25]. The final results were expressed as µmol Trolox equivalent (TE) per g of dried plant. The content of phenolic compounds was investigated by the method by Singleton and Rossi [43] and the total phenolic content (TPC) was expressed as mg GAE (gallic acid equivalent) per g of dried plant.

### 2.3. Preparation of Meat Samples with Plant Extracts

The preparation of meat samples with plant extracts (0.5% m/m) was performed according to the procedure by Muzolf-Panek et al. [25]. Each sample was put in a low-density polyethylene bag and stored at 4 °C for 11 days, at 8 °C and 12 °C for 7 days, and at 16 °C and 20 °C for 5 days in a thermostatically controlled cabinet (Pol-Eco Aparatura, Wodzisław Śląski, Poland). Various storage periods resulted from the rate of protein oxidation. With increasing temperature the oxidation rate increased.

### 2.4. SH Content

Protein oxidation was investigated in terms of changes in SH content, which was measured spectrophotometrically (Cary 1E spectrophotometer, Varian) using Ellman’s reagent (DTNB – 5,5′-Dithiobis(2-nitrobenzoic acid)) [44] via the modified method [8]. The final results were expressed as nmol cysteine per mg of protein. The calibration curves for BSA (bovine serum albumin) in the range of 0–1.5 mg/mL as well as for L-cysteine in the range of 0–100 µM were prepared. 

Since the protein-containing extracts did not absorb light at a wavelength above 300 nm, as was also observed by others [45], myoglobin was found not to interfere with further measurements (at 412 nm). Non phenolic–protein interaction was observed in the samples, nor was any phenolic interaction with DTNB observed. None of the tested samples showed absorbance readings lower than the control, which would indicate the possible hindering of NTB formation from DTNB breakage or pro-oxidant effects.

In order to obtain universal models, SH changes during storage were given in percentages. Day 0 was used as the initial value, with SH equaled to 100%.

### 2.5. Kinetic Arrhenius and Log-Logistic Models

Kinetic models for food-quality loss can be determined based on the following general equation:

(1) $$-dQ/dt=kQ^{n}$$

where *Q* is a quality index; *t* is time; *k* is kinetic constant rate, which is temperature dependent; and *n* is kinetic order [30].

SH changes during storage at constant temperature were modeled by means of a zero-order equation and Equation (1) for *n* = 0 is:

(2) $$SH=SH_{0}-kt$$

where *SH* is the content of thiol groups (%), *SH_0_* is the initial value (100%) at time 0, *k* is the meat-quality rate constant (day^−1^) at a given temperature, and *t* is time (day). SH changes at 4, 8, 16, and 20 °C were used to establish the kinetic models. Linear regression was obtained by plotting SH changes (%) versus time (day).

The effect of temperature on the rate constant (*k*) was calculated according to the Arrhenius equation:

(3) $$k=k_{0}\;exp\left(-E_{a}/RT\right)$$

where *k* (day^−1^) represents the SH content rate, *k*_0_ is the pre-exponential factor, *Ea* (kJ/mol) is the activation energy, *R* is the universal gas constant, and *T* is the absolute temperature.

The linearized form of the Arrhenius equation is:

(4) $$\ln k=\ln k_{0}-E_{a}/RT$$

A plot of ln*k* on the reciprocal of *T* gave the regression line with the slope equal to *–Ea/R* and an intercept of ln*k*_0_. The Arrhenius model used for the prediction of product quality is an empirical rather than physical one. This is because in food temperature dependence is investigated for very complicated reactions and not for defined, simple reactions [46]. 

A log-logistic model was also used to expressed the temperature dependency of food-quality rate constant (*k*):

(5) $$k=m’\ln(1+\exp\left(c\left(T-T_{c}\;\right)\right)$$

where *c* (°C^−1^), *m*’ (−), and *T_c_* (°C^−1^) are empirical fit constants and *m’* = 1 [37]. The difference between Equations (3) and (5) is that model from Equation (5) does not need the concept of activation energy [46].

Finally, the predictive models were obtained by combining Equation (2) and Equation (4) as well as Equation (2) with Equation (5). Values of empirical constants were evaluated using a non-linear estimation analysis by least-squares criterion with the Levenberg-Marquardt algorithm.

Validation was performed on the external dataset (not used for model determination) of SH content in meat stored at 12 °C.

### 2.6. Artificial Neural Networks (ANNs)

ANNs are a data mining tool used in regression and classification analyses. Knowledge of the relations between variables is not required in ANNs, thus they are often called “black box” models. The ability to analyze huge data matrices and to learn based on the training dataset are the main advantages of this tool. ANNs have been applied in food sciences to describe and predict quality changes in food, for process control, and for various simulations [47]

In this study, the dataset was divided into learning (70%), testing (15%), and validating (15%) subsets. Multilayer feed-forward connected ANNs using multilayer perceptron (MLP) and radial basis function (RBF) networks were trained with the Broyden–Fletcher–Goldfarb–Shanno learning algorithm (200 epoch). In total, 20 ANNs were evaluated and the best five were retained. The ANNs consisted of an input layer including 16 neurons, a hidden layer including 4–10 neurons, and an output layer consisting of 1 neuron (SH values as a response). For the learning process, the sums of the squares and the cross-entropy error functions were applied. The success of the ANN models to predict SH content in meat samples was evaluated based on the performance calculated for learning, testing, and validating steps. Performance is defined as a percentage of the samples in the corresponding dataset correctly predicted by the model. Moreover, external validation was performed, which included SH values (%) at 12 °C.

### 2.7. Multiple Linear Regression Models

In order to analyze the effect of plant-extract addition to pork meat on the SH content, slopes of the multiple regression equation representing SH changes with time at constant temperature were compared. 

The general model of MLR is:

(6) $$y=\beta_{0}+\beta_{1}\;x_{1}+\beta_{2}\;x_{2}+\ldots +\beta_{k}x_{k}+\varepsilon ,$$

where *y* is the dependent variable value, *β*_0_ is the intercept, *β*_1−*k*_ are the regression coefficients, *x*_1–*k*_ are the predictors, and *ε* is the standard estimation error. The comparisons between the coefficients were performed by introducing 13 (*k*−1) dummy variables as predictors to the regression analysis. The control samples were not coded since all other categories (13 plant extracts) were compared to the control. 

### 2.8. Statistical Analysis

All results are expressed as mean ± standard deviations calculated for three replications. Statistica 13.3 software (StatSoft, Tulsa, OK, USA) was used at significance level of *p* = 0.05. The determination coefficient (R^2^), root-mean-square error (RMSE), and coefficient of variation (CV%) were used to evaluate the capability of the models. The *t*-test (*p* ≤ 0.05) was used to show the significant differences between the regression coefficients of the samples with plant extract in comparison to the control sample.

## 3. Results and Discussion

### 3.1. Herb and Spice Extracts

The results of the antioxidant activity and phenolic content of the spice and herb extracts are shown in Table S1 and were discussed in the paper by Muzolf-Panek et al. [48]. Since the antioxidant activity of plant extracts was highly correlated with the TPC values (*r* = 0.98, *p* = 0.00) it could be concluded that the antioxidant properties of herbs and spices resulted from these bioactive compound contents. The rank of plant extracts according to the decreasing TPC values was as follows: clove >> allspice > thyme ≥ bay leaf ≈ oregano ≥ basil ≥ onion ≥ rosemary ≈ nutmeg ≈ garlic ≈ black seed ≈ caraway ≈ cardamom. The DPPH^•^ radical scavenging activity decreased in the following order: clove >> allspice > thyme > bay leaf > oregano > basil > rosemary ≥ nutmeg > caraway ≈ garlic ≈ black seed ≈ onion ≈ cardamom. A similar order was reported by Assefa et al. [49] for extracts of selected spices and herbs obtained with 80% methanol. Clove extract was previously reported to have the highest content of phenolics and the highest antioxidant activity [49,50,51].

The phenolic profiles of the spices and herbs tested in the studies have been investigated by others. Among phenolic acids, p-hydroxybenxoic, protocatechuic, caffeic ferulic, rosmarinic, and chlorogenic acids have been determined in bay leaf, nutmeg, onion, oregano, thyme and rosemary, whereas protocatechuic, p-coumaric, caffeic, and rosmarinic acids have been determined in basil [8,15,16,17,18,21,52]. Phenolic acids reported in caraway are as follows: p-hydroxybenzoic, p-coumaric, syringic, caffeic, ferulic, rosmarinic and chlorogenic acids, and in allspice and cardamom they include protocatechuic, sinapic, and chlorogenic acids [8,15]. Gallic, p-coumaric, ferulic, caffeic, and chlorogenic acids were determined in black seed, although other studies found only flavonoids such as kaempferol and quercetin and their derivatives (mainly glycosides). Quercetin was detected in caraway, clove, garlic, nutmeg, onion, oregano, rosemary, and thyme [15,16,18,21,52], whereas kaempferol was found in high quantity in oregano but also in allspice, clove, garlic, onion, cardamom, and onion [8]. Among flavonoids, catechin was a predominant polyphenol in basil and rosemary extracts [18]. According to the literature, it could be stated that considerable variation within the phenolic profiles of selected spices and herbs was observed.

### 3.2. Arrhenius and Log-Logistic Models

Thiol loss is one of the indices providing an evaluation of the extent of protein oxidation and it was reported that free thiols are highly correlated with the content of carbonyl compounds—another marker of protein oxidation [4]. This method has been successfully applied in raw meat [8,25,29], cooked meat, and meat products [26,53,54].

**Figure 1:**
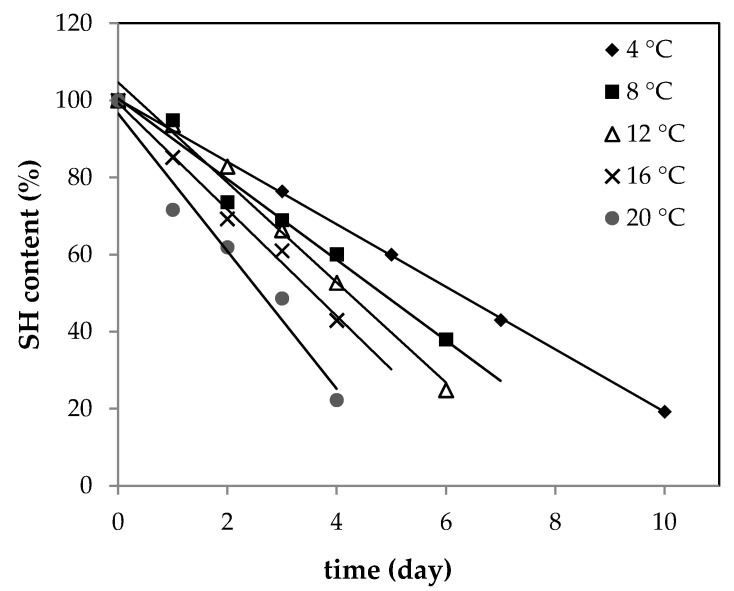
Changes in SH (thiol group) content of raw ground pork (control sample) stored at 4, 8, 12, 16, and 20 °C.

Thus, SH content was determined in raw ground pork samples with plant addition. Samples were stored at controlled temperature conditions during storage time. Figure 1 presents SH changes for the control sample (without any addition). The plot of SH content versus time gave linear regression with a high regression coefficient; therefore, a zero-order reaction model was used. As shown in Figure 1, the content of SH dropped significantly with time and the absolute rate value increased as storage temperature increased. A similar trend was observed previously by Wang et al. in rabbit meat [29]. 

The loss of SH was the most pronounced in the control sample. After the addition of plant extracts to pork meat, the rate of protein oxidation decreased. The absolute values of all rate constants (*k*) derived from kinetic linear regression models are shown in Table 1.

In order to model the temperature dependency of the SH values, Arrhenius as well as log-logistic equations were introduced by combining Equation (2) and Equation (4) as well as Equation (2) and Equation (5), respectively. Both models showed high accuracy of model parameters with similar average values of determination coefficients equal to 0.953 and 0.958 for Arrhenius and log-logistic, respectively. The highest R^2^ values were obtained for the caraway samples for both models (above 0.99), whereas the lowest was for the bay leaf- (R^2^ = 0.84) and cardamom-treated samples (R^2^ = 0.89) for the Arrhenius and log-logistic models, respectively. All model parameters are shown in Table 1.

The *E_a_* value calculated from the Arrhenius equation for the control sample (raw pork meat without plant extract) was equal to 31.2 kJ/mol, and apart from the cardamom-treated sample, it was significantly lower from the samples with plant extracts. Since the SH content was monitored in the meat system, the concept of *E_a_* as the minimum energy required for the reaction should be discussed very carefully, which was mentioned by van Boekel [30]. In this study, *E_a_* values indicated how temperature sensitive the content of SH in each sample was. In this study, *E_a_* values suggested that protein oxidation is less sensitive to temperature in the control sample than in the treated ones (except in the cardamom sample). The most sensitive to temperature were the samples with oregano and basil, with the highest *E_a_* values of 64.8 and 62.3 kJ/mol, respectively. The samples ranged in increasing order of *E_a_* values (from the least sensitive to the most sensitive to temperature) as follows: cardamom < control < onion ≤ bay leaf ≤ black seed < nutmeg ≤ rosemary ≤ allspice ≤ caraway < thyme < garlic < clove < basil < oregano (Table 1).

Since the meat matrix is very complex, it could be only supposed that generally, SH groups are more likely to be oxidized in the control sample than in extract-treated samples, which is in agreement with a previous study [25], but the effect of temperature on the rate of protein oxidation was more visible in the treated samples.

Based on the parameters derived from the Arrhenius model, SH content in ground pork meat with various plant extract additions could be described by the equation:

(7) $$SH=SH_{0}-k_{0}\;\exp\left(-E_{a}/RT\right)\;t$$

where *SH* is the value of the SH index (%), *SH_0_* is the initial value (100%) at time 0, *k*_0_ represents the SH loss rate (Table 1), *E_a_* is the activation energy (Table 1), *R* is the universal gas constant, *T* is absolute temperature, and *t* is the storage time.

Temperature dependency was also introduced by the log-logistic equation and the model was as follows:

(8) $$SH=SH_{0}-\ln\left(1+\exp\left(c\left(T-T_{C}\right)\right)\right)t$$

where *SH* is the value of the SH parameter (%), *SH_0_* is the initial value (100%) at time 0, *c* (°C^−1^) and *T_c_* (°C^−1^) are empirical fit constants, and *t* is the storage time.

To verify the Arrhenius and log-logistic models, the goodness of fit was calculated for the observed versus predicted SH data at 4, 8, 16, and 20 °C and are shown in Table 2. To this end, the adjusted R^2^ values, RMSE, and sum of adjusted R^2^ values were determined. The Arrhenius models showed similar goodness of fit as the log-logistic models, with the mean adjusted R^2^ values equal to 0.952 and 0.945, respectively, and the mean sum of R^2^ values equal to 3.81 and 3.80, respectively. Thus, both models could be applied to describe SH changes at various time/temperature conditions. The highest model accuracy was shown for the pork meat with caraway extract and the lowest for the onion-treated sample. 

### 3.3. ANN Model

Five MLP-ANN models with the highest accuracy are shown in Table 3. The ANNs built for SH content in raw ground pork with extract addition were based on Tanh and exponential functions in the hidden layer, whereas logistic, Tanh, linear, and exponential functions were used in the output layer. Data for all samples (with and without plant extracts) were used to construct ANNs. The accuracy of the networks was very high (above 0.95) in the learning, testing, and validation steps. Based on the values of the adjusted determination coefficient and RMSE, the best network was MLP 16-10-1, with R^2^ = 0.999 and very small RMSE equal to 3.0. 

### 3.4. External Validation of Prediction Models

For all determined models, the external validation was performed using data on SH content in pork samples stored at 12 °C. The observed and predicted SH values from the Arrhenius, log-logistic and MLP-ANNs (the combined five best networks) are presented in Table 4. The verification of the model was discussed based on the determination coefficients, RMSE, and CV values, all calculated from residuals. The highest prediction ability was reported for the log-logistic model (R^2^ = 0.96, RMSE = 5.09, and CV = 4.04). Arrhenius and MLP-ANN showed similar accuracy in predicting SH content in raw pork, with R^2^ equal to 0.948 and 0.944, respectively. Previously, Arrhenius models were developed based on the changes in various indices in food products [29,31,35,36,38,39]. In addition, RBF-ANNs were successfully used for the prediction of quality changes in bream fillets [41], Gouda cheese [55], and shrimp [35].

### 3.5. Effect of Plant Extract Addition Using MLR Model

MLR was performed to assess the effect of plant extracts on SH content in raw pork in various time/temperature conditions. All results of the regression analysis are shown in Table 5. Calculated from MLR, the coefficient (β) for the plant extracts represents the difference in SH content between the control and treated samples. The higher the absolute value of β, the lower the extent of protein oxidation was in the sample. Based on the regression coefficients, all plant extracts slowed down the loss of SH groups in raw pork, thus increasing the oxidative stability of the proteins. 

Oregano and thyme were the most potent inhibitors of thiol oxidation among the extracts studied, although they showed moderate radical scavenging capacity against DPPH^•^. Their effectiveness against SH loss was superior to clove and allspice extracts, which in turn were characterized by the highest TPC values and the antioxidant activity in this study. Since meat is a very complex matrix, the antioxidant activity of the extracts in meat could be affected by many factors, such as presence of other antioxidant active compounds native to meat (enzymes, metal ions, myoglobin), various interactions between the antioxidants from extracts and meat compounds (polyphenol–protein interactions in particular could result in the decrease of the antioxidant activity of the extracts), or the physico-chemical properties of the bioactive compounds in extracts (for example, expressed as logP of the compounds). The effect of optimal concentration of the antioxidant could also have some impact on the results obtained, since it has been shown that too-high or too-low concentrations of the antioxidant in food could induce various effects, even leading to pro-oxidant activity in the case of high concentrations. Thus, even when discussing the phenolic profile of the extracts, it would be not possible to point out which of the determined phenolic compounds or which mechanism could underlie the observed effect. We could only suppose that the effect of oregano extract addition in meat could result from high amounts of kaempferol or carvacrol in oregano extract in comparison to other spices and herbs [18,48,56]. Carvacrol was also detected in high quantities in thyme extract [56].

Nieto et al. [26] reported that oregano essential oils could retard the SH loss in pork patties. The order of selected spices and herbs according their influence on SH content was similar to the previous results [25], with caraway being one of the most active extracts.

## 4. Conclusions

This study establishes various kinetic predictive models for protein oxidation expressed as SH content in raw ground pork after the addition of various plant extracts. The decrease in SH content with time was well described by the zero-order equation. The temperature dependence was adequately modeled and validated by the Arrhenius and log-logistic models. In addition, ANN showed high validation performance. All models showed realistic prediction of data with CV ranging from 4 to 4.5%, which confirmed a low relative dispersion of data around the mean in the modeled dataset. The verification of the implemented models showed the log-logistic model as the one with slightly better accuracy comparing to the Arrhenius and MLP-ANN models. Artificial neural networks usually show greater predictive ability; however, in this research an additional qualitative predictor (extract type) was introduced into the models, whereas in the kinetic models, only time and temperature were introduced as quantitative predictors. Moreover, the influence of selected herbs and spices on the SH content in raw pork was assessed by an MLR model. Oregano, thyme, and caraway were noted as highly effective extracts in lowering protein oxidation in pork. However, no correlation was observed between the radical scavenging activity of the extracts and their activity in meat. Further research should establish compounds responsible for such effects of the extracts, but even when discussing the phenolic profile of the extracts, it would be not possible to point out exactly which of the determined phenolic compounds or which mechanism could underlie the observed effect. This confirms the importance of examining the antioxidant efficacy of plant extracts in a specific food matrix. The high antioxidant activity of an extract alone does not guarantee its high efficacy in particular complex food matrices (such as meat).

To maintain high quality in products during shelf life, the important issue is to have appropriate tools for monitoring quality changes. This could be realized by using predictive kinetic models. However, because of the complexity of the meat a matrix, where various reactions could occur simultaneously and interfere with each other, conclusions on the final product quality should be drawn very carefully. More investigation is needed to obtain full information in this area.

## Figures and Tables

**Table 1:** Estimated kinetic parameters of Arrhenius and log-logistic models for thiol group (SH) content in raw ground pork with plant extracts during storage at 4, 8, 16, and 20 °C.

Extract	Temperature (K)	k (d^−1^)	Arrhenius			Log-Logistic		
			R^2^	*E_a_* (kJ/mol)	*k*_0_ (d^−1^)	R^2^	*c* (°C^−1^)	*T_c_* (°C^−1^)
Control	277	8.1477 ± 0.0814	0.9844 ± 0.0059	31.21 ± 0.46	6.4 × 10^6^ ± 1.2 × 10^6^	0.9695 ± 0.0047	0.5695 ± 0.0029	10.081 ± 0.346
	281	10.4655 ± 0.2743						
	289	13.8307 ± 0.0381						
	293	17.8547 ± 0.1424						
Allspice	277	5.5696 ± 0.1429	0.9573 ± 0.0226	41.08 ± 0.62	3.4 × 10^8^ ± 8.8 × 10 ^7^	0.9767 ± 0.0222	0.5868 ± 0.002	6.001 ± 0.01
	281	8.3583 ± 0.1037						
	289	13.683 ± 0.5107						
	293	14.6404 ± 0.3064						
Basil	277	3.6157 ± 0.1467	0.955 ± 0.009	62.28 ± 0.46	2.3 × 10^12^ ± 4.0 × 10^11^	0.9643 ± 0.0036	0.8323 ± 0.0137	0.311 ± 0.006
	281	7.4565 ± 0.006						
	289	11.96 ± 0.2746						
	293	17.9823 ± 0.2802						
Bay leaf	277	5.0828 ± 0.0143	0.8416 ± 0.0176	36.73 ± 0.66	5.2 × 10^7^ ± 1.4 × 10^7^	0.9011 ± 0.0235	0.4726 ± 0.0115	9.083 ± 0.505
	281	9.314 ± 0.0553						
	289	12.51 ± 0.2037						
	293	12.9375 ± 0.2903						
Black seed	277	6.0303 ± 0.085	0.9983 ± 0.0022	38.08 ± 0.05	9.1 × 10^7^ ± 1.4 × 10^6^	0.9876 ± 0.0039	0.5542 ± 0.0015	6.377 ± 0.023
	281	7.7736 ± 0.0992						
	289	11.884 ± 0.2261						
	293	15.0553 ± 0.0468						
Caraway	277	3.7622 ± 0.1607	0.9983 ± 0.0009	42.53 ± 0.73	4.1 × 10^8^ ± 1.1 × 10^8^	0.9934 ± 0.0001	0.4131 ± 0.0048	4.642 ± 0.246
	281	5.1103 ± 0.1271						
	289	8.2472 ± 0.2125						
	293	10.4214 ± 0.2175						
Cardamom	277	6.8443 ± 0.0288	0.8834 ± 0.0294	23.58 ± 1	2.2 × 10^5^ ± 9.1 × 10^4^	0.8948 ± 0.0207	0.4976 ± 0.2784	14.118 ± 6.698
	281	9.6445 ± 0.1948						
	289	10.5695 ± 0.03						
	293	13.1264 ± 0.2994						
Clove	277	3.4777 ± 0.0574	0.9867 ± 0.0078	58.18 ± 1.6	4.0 × 10^11^ ± 2.6 × 10^11^	0.9711 ± 0.0004	0.6747 ± 0.0362	0.655 ± 0.39
	281	5.825 ± 0.1333						
	289	10.0913 ± 0.3264						
	293	14.7812 ± 0.5409						
Garlic	277	3.2008 ± 0.1237	0.9938 ± 0.0006	55.73 ± 1.14	1.1 × 10^11^ ± 5.1 × 10^10^	0.9866 ± 0.0047	0.5646 ± 0.0017	1.134 ± 0.284
	281	5.0483 ± 0.1234						
	289	9.0686 ± 0.2595						
	293	12.4118 ± 0.0329						
Nutmeg	277	5.2189 ± 0	0.9085 ± 0.0246	40.19 ± 0.03	2.3 × 10^8^ ± 1.6 × 10^6^	0.9649 ± 0.016	0.5544 ± 0.006	6.774 ± 0.192
	281	9.2317 ± 0.4906						
	289	12.5299 ± 0.0444						
	293	14.6567 ± 0.3445						
Onion	277	4.9511 ± 0.3146	0.9576 ± 0.0236	35.34 ± 2.11	2.8 × 10^7^ ± 2.4 × 10^7^	0.9262 ± 0.0099	0.4022 ± 0.0273	7.09 ± 0.883
	281	5.7084 ± 0.4963						
	289	8.352 ± 0.0031						
	293	11.6554 ± 0.4791						
Oregano	277	2.7732 ± 0.1898	0.9273 ± 0.0113	64.76 ± 4.56	1.0 × 10^13^ ± 1.4 × 10^13^	0.9481 ± 0.0088	0.5553 ± 0.0411	0.89 ± 0.768
	281	2.7478 ± 0.1094						
	289	8.8805 ± 0.0506						
	293	10.503 ± 0.4606						
Rosemary	277	5.023 ± 0.2823	0.9835 ± 0.004	40.52 ± 0.7	2.3 × 10^8^ ± 5.6 × 10^7^	0.958 ± 0.0047	0.7291 ± 0.0256	1.847 ± 0.138
	281	6.6476 ± 0.3802						
	289	11.4884 ± 0.6257						
	293	12.651 ± 0.4627						
Thyme	277	3.3421 ± 0.2202	0.9718 ± 0.0057	52.19 ± 2.37	2.8 × 10^10^ ± 2.5 × 10^10^	0.9655 ± 0.0064	0.4781 ± 0.0084	1.399 ± 0.488
	281	3.6951 ± 0.1168						
	289	8.1486 ± 0.0084						
	293	10.5541 ± 0.1328						

*k* (day^−1^) represents the SH content rate, *k*_0_ is the pre-exponential factor, *Ea* (kJ/mol) is the activation energy from the Arrhenius equation, and *c* (°C^−1^) and *T_c_* (°C^−1^) are empirical fit constants from the log-logistic model.

**Table 2:** Accuracy of Arrhenius and log-logistic models of SH content in raw ground pork with various plants extracts added during storage at 4, 8, 16, and 20 °C.

Extract	Temperature (K)	Model					
		Arrhenius			Log-Logistic		
		Adj. R^2^	RMSE	ΣR^2^	Adj. R^2^	RMSE	ΣR^2^
Control	277	0.9979 ± 0.0015	1.08 ± 0.24	3.93	0.9980 ± 0.0018	1.19 ± 0.64	3.93
	281	0.9826 ± 0.0124	3.43 ± 0.35		0.9826 ± 0.0114	3.29 ± 0.35	
	289	0.9896 ± 0.0083	2.36 ± 0.17		0.9896 ± 0.0109	2.83 ± 0.07	
	293	0.9617 ± 0.0257	6.38 ± 0.04		0.9617 ± 0.0289	6.75 ± 0.03	
Allspice	277	0.9788 ± 0.0199	2.28 ± 0.84	3.81	0.9848 ± 0.0150	1.86 ± 0.84	3.81
	281	0.9065 ± 0.0781	6.03 ± 0.80		0.9065 ± 0.0602	5.29 ± 0.71	
	289	0.9670 ± 0.0292	3.97 ± 1.01		0.9670 ± 0.0240	3.73 ± 0.77	
	293	0.9538 ± 0.0366	5.75 ± 0.60		0.9538 ± 0.0424	6.28 ± 0.53	
Basil	277	0.9933 ± 0.0052	1.85 ± 0.30	3.85	0.9876 ± 0.0129	2.04 ± 0.70	3.84
	281	0.9257 ± 0.5989	7.20 ± 0.97		0.9257 ± 0.4616	5.01 ± 0.99	
	289	0.9901 ± 0.0086	2.35 ± 0.71		0.9901 ± 0.0159	3.18 ± 0.60	
	293	0.9415 ± 0.0376	8.64 ± 0.09		0.9415 ± 0.0591	10.75 ± 0.04	
Bay leaf	277	0.9421 ± 0.0496	3.94 ± 0.78	3.79	0.9280 ± 0.0609	4.44 ± 0.77	3.78
	281	0.9299 ± 0.0572	5.73 ± 0.51		0.9299 ± 0.0337	4.34 ± 0.48	
	289	0.9612 ± 0.0319	4.15 ± 0.47		0.9612 ± 0.0273	3.83 ± 0.44	
	293	0.9565 ± 0.0388	4.57 ± 1.05		0.9565 ± 0.0446	4.88 ± 1.16	
Black seed	277	0.9495 ± 0.0418	3.43 ± 0.16	3.84	0.9259 ± 0.0615	4.15 ± 0.21	3.81
	281	0.9478 ± 0.0411	4.76 ± 0.43		0.9478 ± 0.0486	5.19 ± 0.50	
	289	0.9694 ± 0.0239	3.85 ± 0.34		0.9694 ± 0.0271	4.17 ± 0.54	
	293	0.9716 ± 0.0231	4.49 ± 0.65		0.9716 ± 0.0279	4.99 ± 0.65	
Cardamon	277	0.9879 ± 0.0147	1.07 ± 0.35	3.97	0.9927 ± 0.0063	1.11 ± 0.36	3.98
	281	0.9964 ± 0.0035	1.14 ± 0.79		0.9964 ± 0.0074	1.01 ± 0.48	
	289	0.9910 ± 0.0132	1.04 ± 0.63		0.9910 ± 0.0226	1.30 ± 1.03	
	293	0.9989 ± 0.0009	0.90 ± 0.50		0.9989 ± 0.0024	1.13 ± 0.97	
Caraway	277	0.9756 ± 0.0197	2.87 ± 0.34	3.76	0.9765 ± 0.0190	2.82 ± 0.34	3.76
	281	0.9262 ± 0.0557	6.90 ± 0.94		0.9262 ± 0.0572	6.99 ± 0.97	
	289	0.9664 ± 0.0279	3.77 ± 0.78		0.9664 ± 0.0314	3.98 ± 0.78	
	293	0.8946 ± 0.0834	8.23 ± 0.68		0.8946 ± 0.0887	8.49 ± 0.69	
Clove	277	0.9769 ± 0.0208	2.03 ± 0.46	3.80	0.9548 ± 0.0402	2.84 ± 0.63	3.78
	281	0.9787 ± 0.0172	2.63 ± 0.54		0.9787 ± 0.0216	2.95 ± 0.79	
	289	0.9001 ± 0.0842	5.73 ± 0.74		0.9001 ± 0.0631	5.07 ± 0.48	
	293	0.9477 ± 0.0399	6.89 ± 0.54		0.9477 ± 0.0567	8.19 ± 0.68	
Garlic	277	0.8927 ± 0.0918	3.56 ± 0.29	3.74	0.8296 ± 0.1458	4.48 ± 0.37	3.68
	281	0.9531 ± 0.0394	2.91 ± 0.12		0.9531 ± 0.0201	2.07 ± 0.18	
	289	0.9385 ± 0.0512	4.08 ± 0.13		0.9385 ± 0.0466	4.05 ± 0.21	
	293	0.9566 ± 0.0360	4.93 ± 0.67		0.9566 ± 0.0508	5.93 ± 0.70	
Nutmeg	277	0.9799 ± 0.0162	2.57 ± 0.00	3.78	0.9808 ± 0.0155	2.51 ± 0.00	3.78
	281	0.9426 ± 0.0436	5.55 ± 0.55		0.9426 ± 0.0292	4.66 ± 0.63	
	289	0.9065 ± 0.0790	6.50 ± 1.09		0.9065 ± 0.0707	6.11 ± 1.08	
	293	0.9538 ± 0.0398	5.45 ± 1.16		0.9538 ± 0.0499	6.29 ± 1.11	
Onion	277	0.8612 ± 0.1178	4.56 ± 0.40	3.67	0.8142 ± 0.1577	5.26 ± 0.48	3.62
	281	0.9087 ± 0.0896	3.70 ± 0.84		0.9087 ± 0.0844	3.45 ± 0.95	
	289	0.9396 ± 0.0514	3.52 ± 0.29		0.9396 ± 0.0694	4.14 ± 0.26	
	293	0.9590 ± 0.0343	4.64 ± 1.29		0.9590 ± 0.0334	4.58 ± 1.30	
Oregano	277	0.9256 ± 0.0676	2.73 ± 0.71	3.79	0.8044 ± 0.1707	4.21 ± 0.70	3.67
	281	0.9281 ± 0.0659	2.16 ± 0.45		0.9281 ± 0.1448	3.34 ± 0.50	
	289	0.9629 ± 0.0313	3.43 ± 0.60		0.9629 ± 0.0101	2.04 ± 0.70	
	293	0.9717 ± 0.0231	3.45 ± 0.12		0.9717 ± 0.0182	2.89 ± 0.35	
Rosemary	277	0.9493 ± 0.0426	3.15 ± 0.21	3.76	0.9333 ± 0.0562	3.57 ± 0.28	3.74
	281	0.8712 ± 0.1073	5.98 ± 0.48		0.8712 ± 0.0871	5.46 ± 0.32	
	289	0.9627 ± 0.0318	3.73 ± 0.60		0.9627 ± 0.0236	3.23 ± 0.50	
	293	0.9766 ± 0.0206	3.96 ± 1.26		0.9766 ± 0.0155	3.49 ± 1.09	
Thyme	277	0.8941 ± 0.1013	2.84 ± 0.64	3.82	0.8021 ± 0.1815	3.88 ± 0.81	3.73
	281	0.9727 ± 0.0233	1.66 ± 0.07		0.9727 ± 0.0383	2.16 ± 0.23	
	289	0.9616 ± 0.0326	2.77 ± 0.11		0.9616 ± 0.0507	3.46 ± 0.12	
	293	0.9925 ± 0.0066	1.64 ± 0.32		0.9925 ± 0.0105	2.07 ± 0.40	

Adj. R^2^ is adjusted determination coefficient; RMSE is root mean square error; ΣR^2^ is sum of determination coefficients

**Table 3:** ANN (artificial neural network) model parameters for SH content in raw ground pork enriched with plant extracts stored at 4, 8, 16, and 20 °C.

Net Parameters	Net Structure				
	MLP 16-4-1	MLP 16-4-1	MLP 16-10-1	MLP 16-7-1	MLP 16-10-1
Learning accuracy	0.9712	0.9726	0.9728	0.9823	0.9855
Testing accuracy	0.9687	0.9740	0.9746	0.9768	0.9840
Validation accuracy	0.9582	0.9642	0.9599	0.9750	0.9770
Training error	8.2836	7.8646	7.7707	5.0906	4.1810
Test error	11.2108	9.4961	8.9631	8.1859	5.6804
Validation error	12.1337	10.4650	11.5884	7.2553	6.6660
Training algorithm	BFGS 77	BFGS 68	BFGS 52	BFGS 195	BFGS 131
Error function	SOS	SOS	SOS	SOS	SOS
Hidden activation	Exponential	Tanh	Tanh	Tanh	Tanh
Output activation	Logistic	Tanh	Linear	Exponential	Logistic
R^2^	0.9974	0.9975	0.9977	0.9984	0.9986
RMSE	4.11	4.05	3.90	3.25	3.00

MLP—multilayer perceptron.

**Table 4:** Observed and predicted values of SH content in raw ground pork enriched with plant extract during storage at 12 °C.

Extract	Storage Time (day)	Observed Values	Arrhenius Model	Log-Logistic Model	MLP-ANN Models
Control	0	100.00	100.00	100.00	99.33
	1	93.63	87.88	87.43	88.46
	2	82.86	75.75	74.85	74.21
	3	66.33	63.63	62.28	61.84
	4	52.72	51.51	49.70	50.61
	6	24.81	27.26	24.55	29.79
Allspice	0	100.00	100.00	100.00	99.29
	1	94.86	90.09	89.44	89.42
	2	73.63	80.18	78.88	75.80
	3	68.95	70.27	68.31	64.39
	4	60.07	60.36	57.75	55.06
	6	37.88	40.55	36.63	41.00
Basil	0	100.00	100.00	100.00	95.53
	1	94.48	91.16	89.75	82.30
	2	80.79	82.32	79.51	71.09
	3	71.58	73.49	69.26	63.44
	4	66.01	64.65	59.01	56.67
	6	43.97	46.97	38.52	41.60
Bay leaf	0	100.00	100.00	100.00	100.01
	1	93.65	90.57	90.04	90.38
	2	77.88	81.15	80.08	77.49
	3	64.10	71.72	70.12	66.60
	4	56.27	62.29	60.16	57.44
	6	30.34	43.44	40.23	42.39
Black seed	0	100.00	100.00	100.00	99.50
	1	92.89	90.35	89.82	91.24
	2	80.05	80.71	79.63	80.26
	3	71.70	71.06	69.45	70.40
	4	61.11	61.42	59.26	61.04
	6	43.75	42.13	38.89	42.04
Caraway	0	100.00	100.00	100.00	100.19
	1	92.95	93.57	93.12	94.95
	2	86.32	87.14	86.25	87.88
	3	79.74	80.71	79.37	81.18
	4	73.33	74.28	72.49	74.70
	6	61.49	61.42	58.74	61.21
Cardamom	0	100.00	100.00	100.00	100.75
	1	94.69	90.17	89.95	94.03
	2	83.61	80.34	79.91	84.69
	3	75.09	70.51	69.86	74.05
	4	65.17	60.68	59.82	62.98
	6	42.01	41.02	39.72	41.13
Clove	0	100.00	100.00	100.00	99.68
	1	86.80	92.50	91.47	91.97
	2	80.12	85.00	82.94	82.89
	3	68.84	77.49	74.41	75.02
	4	62.59	69.99	65.88	67.98
	6	45.78	54.99	48.82	55.41
Garlic	0	100.00	100.00	100.00	99.24
	1	90.31	93.40	92.58	91.99
	2	79.25	86.79	85.17	82.88
	3	74.82	80.19	77.75	75.88
	4	70.41	73.58	70.34	70.54
	6	54.13	60.37	55.51	60.38
Nutmeg	0	100.00	100.00	100.00	99.82
	1	90.49	90.22	89.59	89.32
	2	78.77	80.45	79.18	75.74
	3	66.71	70.67	68.77	64.37
	4	52.19	60.89	58.36	54.90
	6	31.08	41.34	37.55	39.34
Onion	0	100.00	100.00	100.00	99.14
	1	93.09	92.71	92.34	93.40
	2	86.03	85.42	84.68	85.17
	3	72.82	78.14	77.01	77.76
	4	71.80	70.85	69.35	71.03
	6	52.70	56.27	54.03	56.89
Oregano	0	100.00	100.00	100.00	99.33
	1	95.52	94.77	93.85	94.41
	2	93.48	89.54	87.69	88.29
	3	82.18	84.31	81.54	82.86
	4	80.58	79.08	75.39	77.80
	6	61.29	68.62	63.08	67.71
Rosemery	0	100.00	100.00	100.00	99.33
	1	94.15	91.59	91.05	90.19
	2	82.97	83.18	82.10	78.16
	3	79.21	74.77	73.15	69.60
	4	64.46	66.36	64.20	63.01
	6	42.22	49.54	46.30	50.37
Thyme	0	100.00	100.00	100.00	100.56
	1	95.51	94.23	93.59	95.26
	2	93.51	88.46	87.19	88.58
	3	91.51	82.70	80.78	82.85
	4	81.33	76.93	74.38	77.29
	6	69.08	65.39	61.57	64.46
R^2^			0.948	0.957	0.944
RMSE			5.618	5.090	5.849
CV (%)			4.422	4.037	4.592

MLP-ANN—multilayer perceptron artificial neural network.

**Table 5:** The results of the multiple linear regression analysis (MLR).

Independent Variables and Intercept	Regression Coefficients (β)	*p*-Values
Oregano	15.69	7.5 × 10^−38^
Thyme	15.49	5.7 × 10^−37^
Caraway	13.75	6.99 × 10^−30^
Onion	11.05	3.04 × 10^−20^
Garlic	10.01	5.48 × 10^−17^
Clove	7.39	4.82 × 10^−10^
Rosemary	7.31	7.28 × 10^−10^
Cardamon	6.34	8.84 × 10^−08^
Black seed	5.63	1.98 × 10^−06^
Bay leaf	4.66	8.00 × 10^−05^
Allspice	4.08	5.43 × 10^−04^
Nutmeg	3.22	6.38 × 10^−03^
Basil	2.43	3.92 × 10^−02^
Time	−8.10	0.00
Temperature	−1.28	9.57 × 10^−160^
Intercept	105.39	0.00

## Data Availability

The data presented in this study is available upon reasonable request.

## Supplementary Materials

The following are available online at https://www.mdpi.com/article/10.3390/antiox10060917/s1, Table S1: Antioxidant activity and phenolic compound content of 50% aqueous ethanol extracts of herbs and spices.
