# Supplementary material for: Predictive Modeling of Thiol Changes in Raw Ground Pork as Affected by 13 Plant Extracts—Application of Arrhenius, Log-logistic and Artificial Neural Network Models
Source: Antioxidants (Basel). 2021 Jun 5;10(6):917. doi: 10.3390/antiox10060917 (PMC8229620; doi:10.3390/antiox10060917)
Supplement: Supplementary file 1 [file antioxidants-10-00917-s001.zip › antioxidants-1184420-supplementary.pdf]

Table S1. Antioxidant activity and phenolic compound content of 50% aqueous ethanol extracts of herbs and spices (all data was from [48] and (*) data from [25])

	DPPH µM TE/g	TPC mg GAE g/DW
Allspice*	555±24^{g}	31.61±0.81^{e}
Basil	134.7±2.3^{c}	14.81±0.35^{bc}
Bay leaf*	231.9±1.5^{e}	22.56±0.16^{cd}
Black seed*	7.59±0.84^{a}	2.46±0.61^{a}
Cardamom*	5.45±0.35^{a}	1.24±0.01^{a}
Caraway*	20.2±0.6^{a}	2.39±0.14^{a}
Clove*	1443±1^{h}	167.2±9.3^{f}
Garlic	14.8±1.6^{a}	3.6±0,05^{a}
Nutmeg*	22.22±0.15^{ab}	3.89±0.14^{a}
Onion	5.74±0.28^{a}	7.05±0.58^{ab}
Oregano	171.6±5.8^{d}	20.7±0.1^{cd}
Rosemary	50.4±3.6^{b}	4.66±0.36^{a}
Thyme	278.3±16.2^{f}	23.5±0.6^{d}

All values are mean ± SD of the three replicates. TPC–total polyphenol content.
$^{(a-h)}$means with the same superscript within the same column are not different (Tukey's test, $p > 0.05$).
